# Monitoring treatment efficacy and resistance in breast cancer patients via circulating tumor DNA genomic profiling

**DOI:** 10.1002/mgg3.1079

**Published:** 2019-12-23

**Authors:** Zhanhong Chen, Tian Sun, Ziyan Yang, Yabing Zheng, Ruoying Yu, Xue Wu, Junrong Yan, Yang W Shao, Xiying Shao, Wenming Cao, Xiaojia Wang

**Affiliations:** ^1^ Department of Medical Oncology Zhejiang Cancer Hospital Hangzhou China; ^2^ Translational Medicine Research Institute Geneseeq Technology Inc Toronto Ontario Canada; ^3^ The Second Clinical Medical College of Zhejiang Chinese Medical University Hangzhou China; ^4^ Nanjing Geneseeq Technology Inc. Nanjing China; ^5^ School of Public Health Nanjing Medical University Nanjing China

**Keywords:** breast cancer, chemotherapy, ctDNA, drug resistance, ERBB2, trastuzumab

## Abstract

**Background:**

One of the major challenges in managing invasive breast cancer (BC) is the lack of reliable biomarkers to track response. Circulating tumor DNA (ctDNA) from liquid biopsy, as a candidate biomarker, provides a valuable assessment of BC patients. In this retrospective study, we evaluated the utility of ctDNA to reflect the efficacy of treatment and to monitor resistance mechanisms.

**Methods:**

Targeted next‐generation sequencing (NGS) of 416 cancer‐relevant genes was performed on 41 plasma biopsy samples of 19 HER2+ and 12 HER2‐ BC patients. Longitudinal ctDNA samples were analyzed in three BC patients over the treatment course for detecting acquired mutations.

**Results:**

In HER2+ BC patients, *ERBB2* somatic copy numbers in ctDNA samples were significantly higher in patients progressed on HER2‐targeted therapy than those who were still responding to the treatment. Recurrent acquired mutations were detected in genes including *ERBB2, TP53, EGFR, NF1*, and *SETD2*, which may contribute to trastuzumab resistance. In longitudinal analyses, the observed mutation allele frequencies were tracked closely in concordance with treatment responses. A novel *ERBB2* p.(Leu869Arg) mutation was acquired in one patient upon resistant to trastuzumab therapy, which was further validated as an oncogenic mutation in vitro and contributed to resistance. In HER2‐ BC patients with chemotherapy resistance, genetic alterations on *TP53, PIK3CA,* and DNA damage repair genes were frequently observed.

**Conclusions:**

In summary, ctDNA monitoring, particularly longitudinal analyses, provides valuable insights into the assessment of targeted therapy efficacy and gene alterations underlying trastuzumab resistance and chemotherapy resistance in HER2+ and HER2‐ BC patients, respectively.

## Graphical abstract

ctDNA molecular alterations in HER2+ patients developed acquired resistance to trastuzumab and Longitudinal analyses of multiple somatic mutations in ctDNA in patients with acquired resistance to trastuzumab.

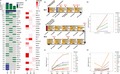

## 1 INTRODUCTION

Breast cancer (BC) is the most frequent cancer among women worldwide including China (Fan et al., 2014), impacting 2.1 million women each year, and also causes the greatest number of cancer‐related deaths among women (WHO data). BC can be classified into different subtypes based on the expression level of human epidermal growth factor receptor (*EGFR*) 2 (*HER2* or *ERBB2*, OMIM 164870) and hormone receptor (HR; Perou et al., 2000; Sørlie et al., 2001).

HER2 is a transmembrane receptor tyrosine kinase (RTK) and is overexpressed in 25%–30% of BC (Geyer et al., 2006; Slamon et al., 2001). These HER2+ BC patients can be treated with HER2‐targeted therapies (Slamon et al., 2001); however, a significant fraction of these patients eventually relapse or develop progressive diseases (Rexer & Arteaga, 2012), which has been reported to be associated with alterations in multiple genes including *PIK3CA* (OMIM 171834)*, PTEN* (OMIM 601728)*, RB1* (OMIM 614041)*, TP53* (OMIM 191170)*, BRCA1* (OMIM 113705)*, BRCA2* (OMIM 600185)*, MAP2K4* (OMIM 601335)*,* and *MAP3K1* (OMIM 600982; Ahmad, Gupta, Kumar, Varshney, & Raghava, 2014). Similarly, HR+ (i.e., estrogen receptor (ER)‐ and/or progesterone receptor‐positive) BC patients have been found to develop resistance to hormonal therapies through *ESR1* (OMIM 133430) mutations (Fanning et al., 2016; Osborne & Schiff, 2011). Lastly, patients with HER2‐/HR‐ BC are referred to as triple‐negative BC (TNBC) and these patients are usually treated with chemotherapies (Fitzmaurice et al., 2017).

There are urgent needs to identify genetic alterations that are important for cancer treatment and drug resistance in order to provide more personalized anticancer therapy, and the development of next‐generation sequencing (NGS) technology enables rapid characterization of mutation profiles of tumor samples. Tumor biopsies are sometimes difficult to obtain and may not represent the genetic heterogeneity of the solid tumor, while circulating tumor DNA (ctDNA), which is released by primary and metastatic tumor cells, has been shown as a noninvasive liquid biopsy for identifying tumor genomic alterations, monitoring tumor progression, and tracking patient's response to treatment (Murtaza et al., 2013; Shu et al., 2017). In BC, it has been reported that ctDNA can be detected in the majority of the patients with early‐ or late‐stage disease, and it correlates with changes in tumor burden and predicts the prognosis (Beaver et al., 2014; Dawson et al., 2013). About 30% of BC patients had detectable *ESR1* mutations in blood after endocrine therapies, and several groups showed that *ESR1* mutations in ctDNA samples were associated with treatment resistance and aggressive clinical behavior in these patients (Chandarlapaty et al., 2016; Gerratana et al., 2019; Schiavon et al., 2015). In addition, longitudinally monitoring *PIK3CA* mutation levels in ctDNA have been found to predict patient progression‐free survival (PFS) in ER+ metastatic BC patients (Gerratana et al., 2019), implying the great clinical values of using ctDNA for diagnosis and prognosis of BC.

In this study, we performed NGS targeting of 416 cancer‐related genes for comprehensive genomic profiling of plasma ctDNAs from 31 BC patients. Overall, our data suggest that plasma ctDNA genomic profiling provides valuable information about treatment efficacy and resistance in BC patients.

## 2 METHODS AND MATERIALS

### 2.1 Ethical compliance

This study was approved by the ethics committee of the Zhejiang Cancer Hospital of Zhejiang Chinese Medical University.

### 2.2 Patient cohort and sample collection

A total of 31 BC patients were included from the Zhejiang Cancer Hospital of Zhejiang Chinese Medical University in this study. Written consent was collected from each patient according to ethical regulations. About 5–10 ml peripheral blood was collected from each patient in EDTA‐coated tubes (BD Biosciences). Plasma was extracted within 2 hr of blood collection and shipped to the central testing laboratory within 48 hr. Formalin‐fixed paraffin‐embedded (FFPE) tumor tissue blocks were obtained from the hospital, with confirmation by the pathologists for diagnosis and tumor purity. Receptor status evaluations were performed by IHC or FISH assays in the pathology department of Zhejiang Cancer Hospital.

### 2.3 DNA extraction, target capture, and next‐generation sequencing

The NGS tests were performed in a centralized clinical testing center (Nanjing Geneseeq Technology Inc.) according to protocols reviewed and approved by the ethical committee of Zhejiang Cancer Hospital. DNA extraction, sequencing library preparation, and targeted capture enrichment were carried out following the methods as previously described with modifications (Shu et al., 2017). Briefly, genomic DNA from whole blood was extracted using the DNeasy Blood & Tissue kit (Qiagen) following the manufacturer's instruction. Plasma sample was first centrifuged at high speed to remove any cell debris, followed by cell‐free DNA (cfDNA) extraction from the supernatant using QIAamp Circulating Nucleic Acid Kit (Qiagen). FFPE samples were deparaffinized with xylene and DNA was extracted using the QIAamp DNA FFPE Tissue Kit (Qiagen) according to the manufacturer's protocols. Sequencing libraries were prepared using the KAPA Hyper Prep Kit (KAPA Biosystems) according to the manufacturer's protocol. Indexed DNA libraries were pooled together and subjected to probe‐based hybridization with GeneseeqOne NGS panel targeting exons of 416 cancer‐relevant genes and introns of 16 fusions genes. Captured libraries were amplified with Illumina p5 and p7 primers in KAPA HiFi HotStart ReadyMix (KAPA Biosystems), and sequenced on Illumina HiSeq4000 NGS platforms (Illumina) according to the manufacturer's instructions. The sequencing assay sensitivity is validated to be over 95%; the limit of detection for single‐nucleotide variant (SNV) and Indel is 2% variant allele frequency across the panel coverage and the limit of detection for SV is 5% variant allele frequency (Jin et al., 2018; Yang et al., 2018).

### 2.4 Sequencing data analysis

Trimmomatic was used for FASTQ file quality control (Bolger, Lohse, & Usadel, 2014). Leading/trailing low quality (quality reading below 30) or N bases were removed. Reads from each sample were mapped to the reference hg19 (Human Genome version 19) using Burrows‐Wheelers Aligner (BWA‐mem v0.7.12; Li & Durbin, 2009). Local realignment around indels and base quality score recalibration were applied with the Genome Analysis Toolkit (GATA 3.4.0).

VarScan2 was employed for detection of candidate somatic mutations in tissue and cfDNA samples (Koboldt et al., 2012). Copy number variations (CNVs) were detected using ADTEx with default parameters (Carter et al., 2012). Somatic CNVs were identified using paired normal/tumor samples for each patient and cutoff was set to 1.6 for copy number gain and 0.6 for copy number loss. Genes that were analyzed and/or discussed include: *PIK3CA* (NM_006218.2), *PTEN* (NM_000314.4), *RB1* (NM_000321.2), *TP53* (NM_001126112.2), *BRCA1* (NM_007294.3), *BRCA2* (NM_000059.3), *MAP2K4* (NM_003010.3), *MAP3K1* (NM_003954.3), *ESR1* (NM_001122742.1), *SETD2* (NM_014159.6), *CDK12* (NM_016507.2), *ERBB2* (NM_004448.2), *EGFR* (NM_005228.3), *NF1* (NM_001042492.2), *PTCH1* (NM_000264.3), *RNF43* (NM_017763.4), *PAK3* (NM_001128166.1), *CDKN1C* (NM_000076.2), *CREBBP* (NM_004380.2), *MLH1* (NM_000249.3), *ARAF* (NM_001654.4), *CRKL* (NM_005207.3), *PRF1* (NM_001083116.1), *DDR2* (NM_001014796.1), *KMT2B* (NM_014727.1), *NRAS* (NM_002524.4), *XPC* (NM_004628.4), and *BUB1B* (NM_001211.5).

## 3 RESULTS

### 3.1 Patients' overview

In this retrospective study, 31 BC patients were included, and the demographic and clinical features of these patients were summarized in Table 1 with details in Table S1. All the BC patients were female with the median age of 47 at diagnosis. Seven patients (22.58%) were diagnosed below age 40, which is considered as early onset BC. Two (BC5 and BC19) of these seven early onset BC patients had *BRCA* germline mutation. Twenty‐six (83.87%) patients received mastectomy. Majority of patients (90.32%) had invasive ductal carcinoma (IDC) at different clinical stages. Of these 31 BC patients, 19 patients were HER2+/HR± and all of them received HER2‐targeted drug trastuzumab and/or lapatinib together with chemotherapy, except for one (BC31) who received hormonal therapy plus chemotherapy. Nine patients were HER2‐/HR+, six of which received chemotherapy only, while the other three received hormonal therapy plus chemotherapy. Three patients were HER2‐/HR‐ (TNBC) and received chemotherapy only.

**Table 1:** The clinicopathological characteristics of all BC patients

Characteristics	Subgroup	No. of patients	Percentage of patients
Gender	Female	31	100.00
	Male	0	0.00
Age at diagnosis	Range	21–64	
	Median	47	
	≥40 year	24	77.42
	<40 year	7	22.58
Mastectomy	Yes	26	83.87
	No	5	16.13
Clinicopathological features	IDC	28	90.32
	Neuroendocrine BC	1	3.23
	“pure” mucinous carcinomas	1	3.23
	Secretory Carcinoma of the Breast	1	3.23
Clinical stage (TNM staging)	I	4	12.90
	II	8	25.81
	III	10	32.26
	IV	6	19.35
	Unknown	3	9.68
HER2 and HR status	HER2+ HR‐	11	35.48
	HER2 + HR+	8	25.81
	HER2‐HR‐	3	9.68
	HER2‐HR+	9	29.03
Treatment	Trastuzumab + Chemotherapy	18	58.06
	Hormonal therapy + Chemotherapy	4	12.90
	Trastuzumab + Hormonal therapy + Chemotherapy	1	3.23
	Chemotherapy only	8	25.81

Abbreviation: BC ,Breast cancer; HR, Hormone receptor; IDC, Invasive ductal carcinoma.

### 3.2 ctDNA profiling correlates with treatment efficacy and disease progression in BC patients

**Figure 1:**
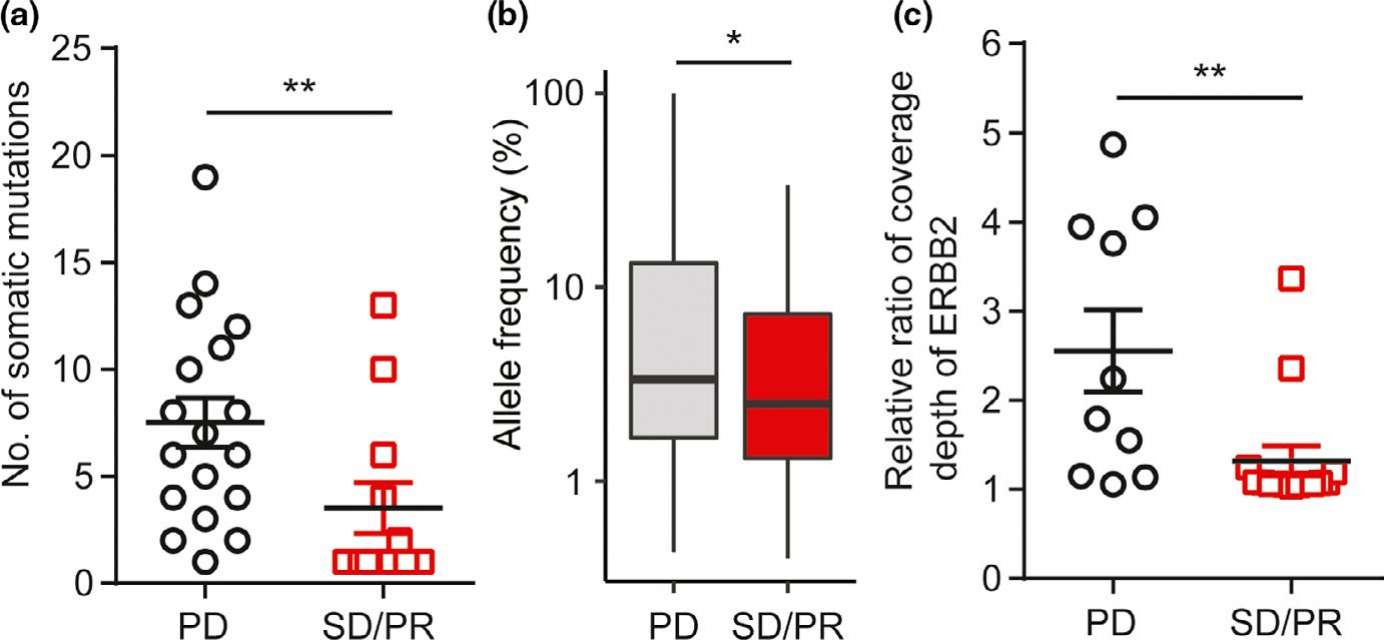
Plasma ctDNA mutation profiling reflects the efficacy of antitumor drugs. (a) The number of somatic mutations in ctDNA in PD versus *SD*/PR BC patients. (b) Allele frequency of all somatic mutations detected in ctDNA in PD versus *SD*/PR BC patients. (c) The relative ratio of coverage depth of *ERBB2* in ctDNA from HER2+ BC patients at PD versus *SD*/PR stage following HER2‐targeted drug therapy. Two‐sided *p* values of less than .05 was considered as statistically significant (*p < .05 and **p < .01). PD: progressed disease; PR: partial response; *SD*: stable disease

It has been previously reported that plasma ctDNA can be detected in early stage BC patients (Beaver et al., 2014), so we performed NGS test on plasma samples obtained from the 31 BC patients (Table S2). First, we investigated if ctDNA can reflect the efficacy of targeted therapy in HER2+ BC patients and chemotherapy in HER2‐ BC patients. We found that the total number of somatic mutations and median of all somatic mutation allele frequency detected in plasma ctDNA were significantly higher in BC patients at the progressed disease (PD) stage compared to those at stable disease (*SD*) or PR stage (Figure 1a,b). Moreover, *ERBB2* relative copy numbers were significantly lower in HER2+ BC patients who benefited from trastuzumab (PR or *SD*) than those developed resistance to trastuzumab (PD; Figure 1c). These data imply that ctDNA profiling might be a valuable tool to monitor disease progression and treatment efficacy.

### 3.3 Genomic profiling of ctDNA from HER2+ BC patients with resistance to trastuzumab

**Figure 2:**
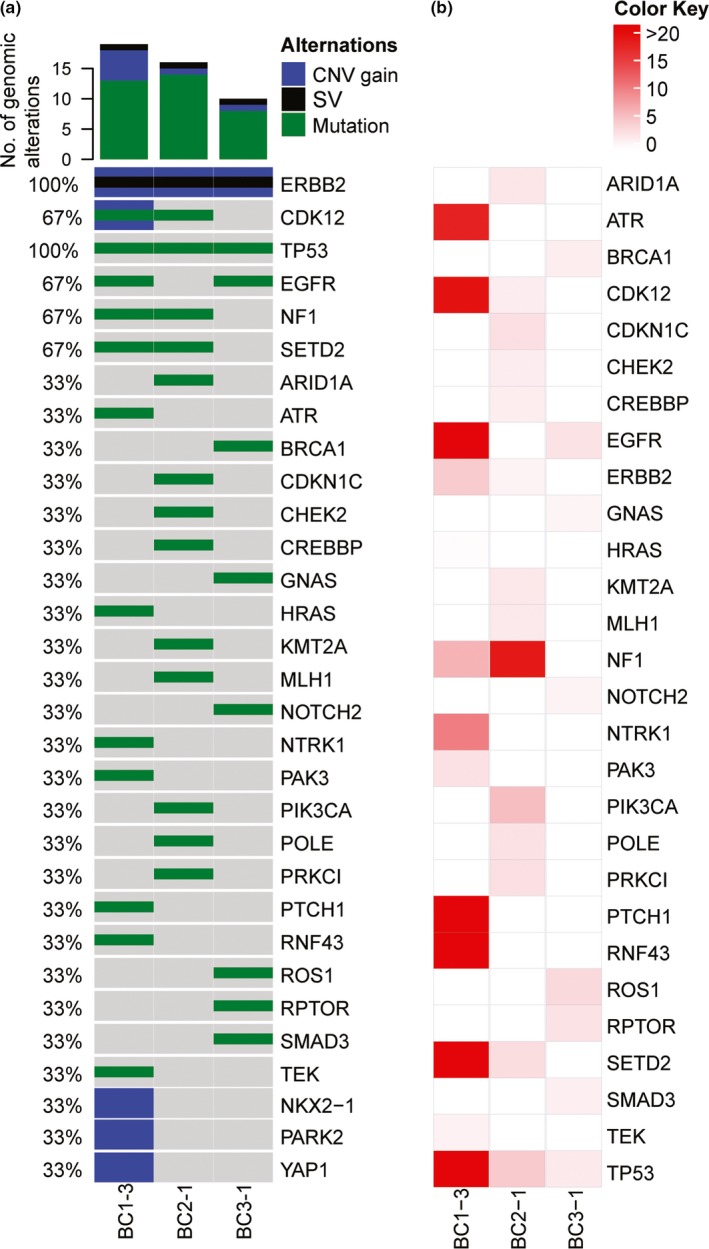
ctDNA molecular alterations in HER2+ patients developed acquired resistance to trastuzumab. (a) The landscape of detected molecular alterations (CNV gain, SV, and mutation) in patients' plasma. (b) Heatmap of allele frequencies of the somatic mutations indicated in Figure 2a. CNV: copy number variation; SV: structural variation

In HER2+ BC patients who progressed on trastuzumab, three patients developed acquired resistance (AR, PFS > 3 months) while three patients had innate resistance (IR, PFS < 3 months). The genetic alteration profiling of the AR patients and the allele frequencies of mutated genes were shown in Figure 2. Along with other reports, mutations in *TP53, SETD2* (OMIM 612778)*, CDK12* (OMIM 615514)*, EGFR,* and *NF1* (OMIM 613113) can be detected in most of the patients. We analyzed these genes based on their functions as tumor suppressors versus oncogenes.

**Figure 3:**
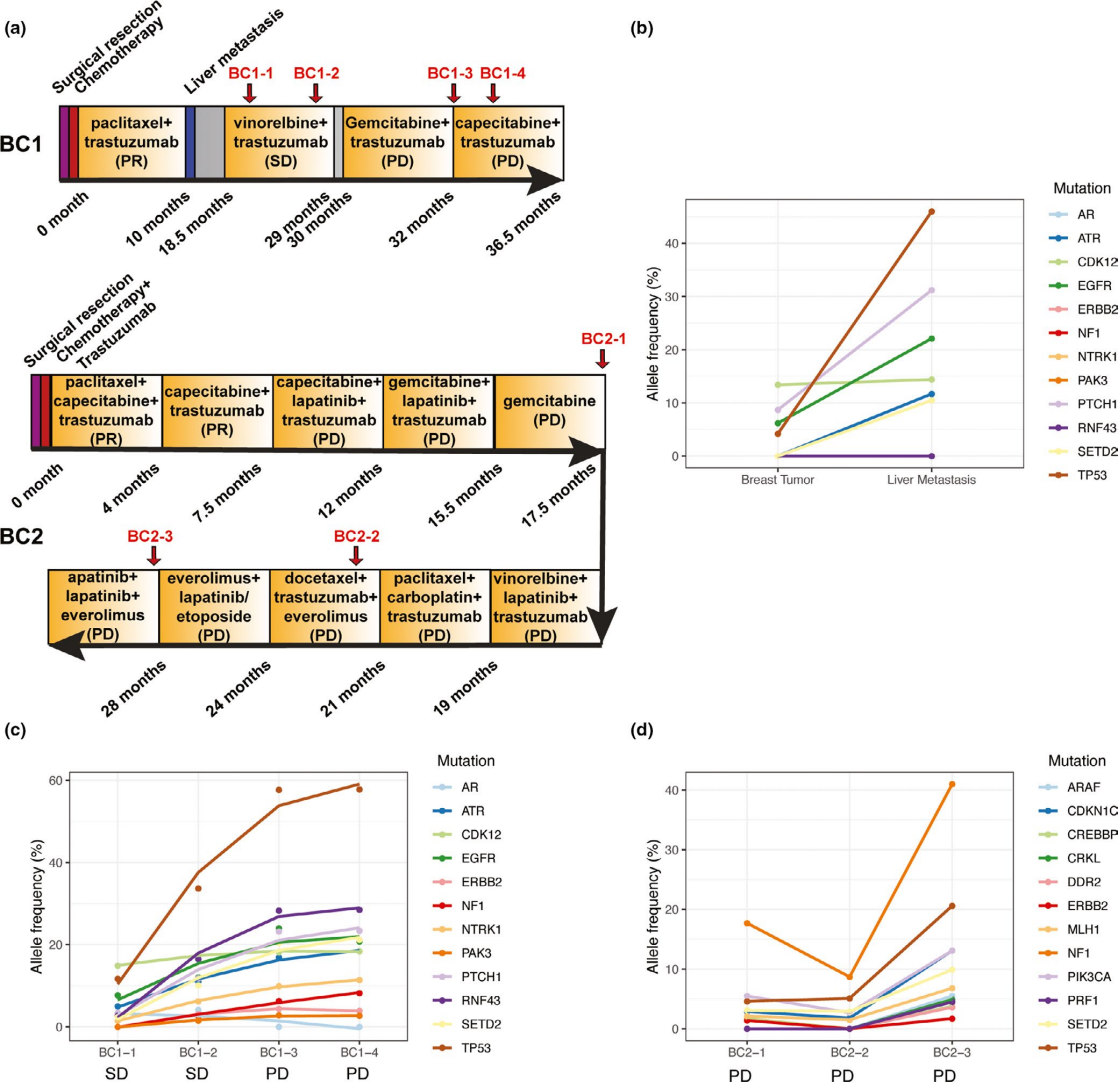
Longitudinal analyses of multiple somatic mutations in ctDNA in patients with acquired resistance to trastuzumab. (a) The clinical history of patients BC1 and BC2. The red arrows indicate the time points of blood sample collection for ctDNA analysis. (b) Allele frequency of mutations identified in the primary breast cancer sample and the liver metastasis sample in patient BC1. (c) Allele frequency of mutations identified in four blood samples in patient BC1. (d) Allele frequency of mutations identified in three blood samples in patient BC2. For all the samples, only somatic mutations with allele frequency >2% were shown

In terms of oncogenes, all three AR patients exhibited *ERBB2* copy number gain and structural variation (SV; Figure 2a). Two of three AR patients had missense somatic mutations in *ERBB2*. Specifically, patient BC1 had *ERBB2* p.(Leu869Arg) mutation in exon 21 while patient BC2 had *ERBB2* p.(Arg1048Cys) mutation in exon 25. The *ERBB2* p.(Leu869Arg) mutation is located within the activation loop of the tyrosine kinase domain and it has been reported to be a gain‐of‐function activating mutation in vitro (Hanker et al., 2017; Wang et al., 2017). The *ERBB2* p.(Arg1048Cys) mutation is in the autophosphorylation tail with unknown variant significance. Since this patient (BC2) had other reported driver mutations, such as a gain‐of‐function *PIK3CA* p.(Cys420Arg; Gymnopoulos, Elsliger, & Vogt, 2007), it is likely that *PIK3CA* p.(Cys420Arg) confers the oncogenicity and drug resistance rather than *ERBB2* p.(Arg1048Cys). Besides *ERBB2*, *EGFR* missense mutations were detected in two patients. Patient BC3 had *EGFR* p.(Arg831His) mutation (exon 21), which is predicted to be a gain‐of‐function mutation (Foster et al., 2010). Patient BC1 had *EGFR* p.(Ala1118Thr) mutation (exon 28) of unknown variant significance. On the other hand, in terms of tumor suppressors, all the *TP53* mutations detected in our AR patients were in the DNA‐binding domain. Patient BC2 exhibited splice acceptor variant in intron 6 (c.673‐1G>A) and patient BC3 had splice donor variant in intron 7 (c.782+1G>T). *TP53* c.782+1G>T mutation has been reported in a lung cancer case (Hu et al., 2013), which is predicted to be pathogenic. *TP53* c.673‐1G>A has been reported in BC patients (Ferrari et al., 2016); however, its implication is unknown. Patient BC1 had a missense mutation *TP53* p.(Cys238Phe), which was observed with increased allele frequencies in the original breast tumor (4.17%), subsequently in the liver metastasis (45.96%), and in serial plasma samples (11.71%‐57.78%; Figure 3). *NF1* is a tumor suppressor through inhibition of RAS activity, whose mutation is frequently found in BC patients (Wallace et al., 2012). *NF1* missense mutation p.(Leu508His) found in BC1 was located in exon 7 with unknown significance. *NF1* missense mutation p.(Ser2817Phe) found in BC2 was located in the last exon of *NF1* (exon 58). This mutation might be deleterious since the mutant allele frequency increased to 41% in the last sampling and was annotated as phosphoserine site in the Uniprot database. *SETD2* is a histone methyltransferase acting as a tumor suppressor by maintaining genome stability (Duns et al., 2010; Pfister et al., 2014). One of the missense p.(Ser1682Leu; exon 9) in patient BC3 was located in the post‐SET domain, which might be deleterious and reduce the genome stability of patient BC3.

In the patients with IR to trastuzumab, we found that one patient had *TP53* p.(Arg175His) hotspot missense mutation (Sigal & Rotter, 2000) at the high allele frequency 29.32%, which confers oncogenicity. One patient had hotspot mutations in *TP53* p.(Arg175His) and *PIK3CA* p.(His1047Arg; Samuels et al., 2004) with low frequencies (1.11% and 0.74%, respectively). The last patient had no detectable ctDNA, but she had a *BRCA*2 N1459S germline mutation, which may confer the IR (Figure S1).

Taken together, our data imply that ctDNA is a valuable approach to detect genetic alterations related to trastuzumab resistance in BC patients, with mutations in DNA damage response pathway and/or PI3K pathway being found in both AR and IR patients while mutations in *HER2* or other RTK/RAS pathway‐related genes being observed more in AR patients. Although some of our observations were supported by previous studies (Asic, 2016), our results were limited by the small patient number and need to be validated in larger BC patient cohorts.

### 3.4 Longitudinal blood sampling and ctDNA mutation profiling can indicate disease progression

To further elucidate the trastuzumab‐resistant process, we collected blood samples longitudinally and monitored the disease progression in the three AR patients (Figure 3a). Patient BC1 was first diagnosed in early stage BC (T1N0M0), with somatic mutations in *TP53, CDK12, EGFR,* and *PTCH1* (OMIM 601309) in the original breast tumor, and these mutations could also be detected in the liver metastasis samples with increased allele frequencies (Figure 3b). Using ctDNA to monitor the disease progression, we found newly emerged somatic mutations with low allele frequencies occurred at the *SD* stage, including *RNF43* (OMIM 612482)*, NTRK1, NF1, ERBB2,* and *PAK3* (OMIM 300142), and the allele frequencies of these mutations dramatically increased when the patient progressed to PD stage (Figure 3c). Patient BC2, after initially benefitted from trastuzumab and lapatinib in combination with chemotherapy treatment, developed PD and AR to both HER2‐targeted drugs. Some of the mutations, such as *NF1, TP53, PIK3CA, CDKN1C* (OMIM 600856)*, SETD2, CREBBP* (OMIM 600140), and *MLH1* (OMIM 120436), were detected in all the serial plasma samples with increased allele frequencies, except for *CREBBP* that showed up in only two samples (Figure 3d). Some mutations, such as *PRKCI, POLE, ARID1A, KMT2A, ERBB2,* and *CHEK2*, were only detected in the first sampling, whereas other mutations, such as *ARAF* (OMIM 311010)*, CRKL* (OMIM 602007)*, PRF1* (OMIM 170280)*, DDR2* (OMIM 191311), and *KMT2B* (OMIM 606834)*,* were only detected in the last sampling (Figure 3d and Table S2). Patient BC3 had low‐frequency mutations when developed PD (data not shown). After changing the treatment, this patient had *SD* and no ctDNA was detectable and thus, no mutation was detected.

In summary, within the limited number of patients, we found that tumor mutation evolution is a dynamic process and multiple oncogenic alterations become more dominant during disease progression; the liquid biopsies seem to give better resolution of the mutated genes in a real‐time manner compared with tumor biopsies, thus providing valuable information regarding disease progression and prognosis prediction. These results need to be confirmed with more patient samples.

### 3.5 *ERBB2* L869R mutation contributes to trastuzumab resistance

**Figure 4:**
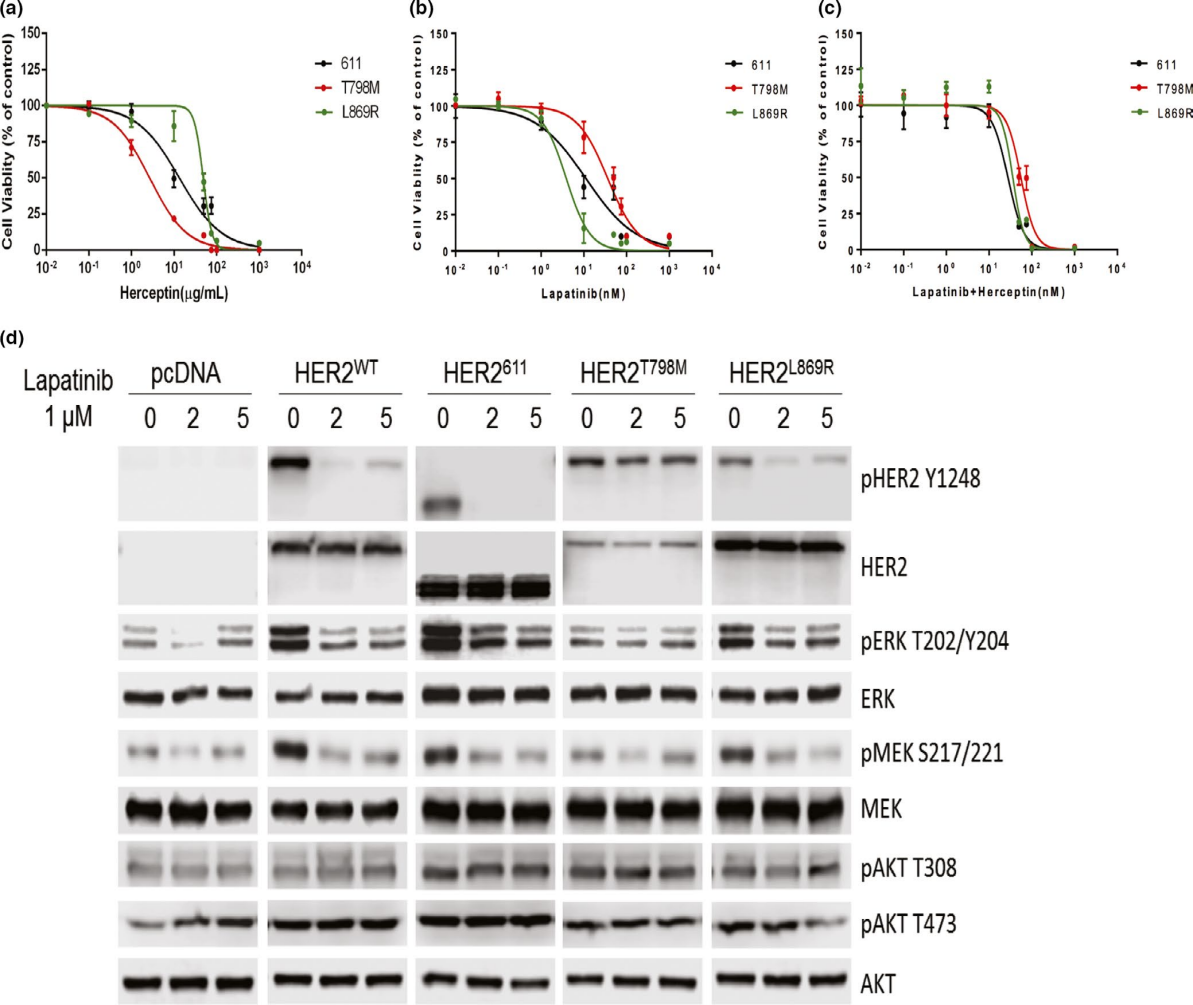
*ERBB2* p.(Leu869Arg) mutation is trastuzumab‐resistant but lapatinib‐sensitive. (a‐c). *ERBB2* p.(Leu869Arg) mutation induces trastuzumab resistance but is sensitive to lapatinib treatment. (d) *ERBB2* p.(Leu869Arg) mutation responses to 1‐μM lapatinib treatment via MEK/ERK downstream pathways, but not PI3K/Akt pathway

As *ERBB2* p.(Leu869Arg) mutation was detected in AR patients, we next functionally investigated whether this mutation contributes to the trastuzumab resistance. We generated mouse Ba/F3 cell lines stably expressing *ERBB2*‐WT, *ERBB2* p.(Leu869Arg), *ERBB2* p.(Thr798Met), and *ERBB2* 611 mutation individually. *ERBB2* p.(Thr798Met) is analogous to *EGFR* p.(Thr790Met) that is shown to cause resistance toward EGFR inhibitors (Kancha et al., 2011). *ERBB2* 611 truncating mutation is oncogenic and resistant to anti‐HER2 therapies (Scaltriti et al., 2007). All *ERBB2* mutants were expressed at comparable levels to the wild‐type *ERBB2* control (Figure S2a). All Ba/F3 cells expressing these *ERBB2* mutants, but not wild‐type *ERBB2* or vector‐only controls, achieved IL3‐independent proliferation (Figure S2b). Next, we exposed the cells carrying the *ERBB2* mutations to increased doses of lapatinib only, trastuzumab only, and lapatinib in combination with trastuzumab (Figure 4a‐c). Compared to the *ERBB2* p.(Thr798Met) mutant, cells carrying the *ERBB2* p.(Leu869Arg) mutation were resistant to trastuzumab while sensitive to lapatinib (Figure 4a,b). The presence of both trastuzumab and lapatinib did not confer additional benefits in these mutants compared to single‐drug treatment (Figure 4c), suggesting if patients carrying *ERBB2* p.(Thr798Met) develop resistance to trastuzumab or patients carrying *ERBB2* p.(Leu869Arg) develop resistance to lapatinib, they may not benefit from additional usage of lapatinib or trastuzumab, respectively.

Given that trastuzumab‐resistant *ERBB2* p.(Leu869Arg) mutation is sensitive to lapatinib (Figure 4b), we further investigated which EGFR downstream signaling pathway was affected. We expressed these mutations in HEK 293T cell lines and exposed the cells carrying the *ERBB2* mutations to 1‐μM lapatinib for 2 and 5 hr. As shown in Figure 4d, *ERBB2* p.(Leu869Arg) mutation was sensitive to lapatinib treatment, compared to *ERBB2* p.(Thr798Met) mutation. Moreover, the MEK/ERK pathway downstream of EGFR signaling pathway was reduced in *ERBB2* p.(Leu869Arg) mutant compared to *ERBB2* p.(Thr798Met) mutant. The PI3K/AKT pathway downstream of EGFR signaling pathway was not affected in all mutants. In summary, these data provide evidence for *ERBB2* p.(Leu869Arg) as a potential trastuzumab‐resistant gene, which is of clinical relevance.

### 3.6 HER2‐ BC patients developed PD after chemotherapy display different somatic mutation profiles

**Figure 5:**
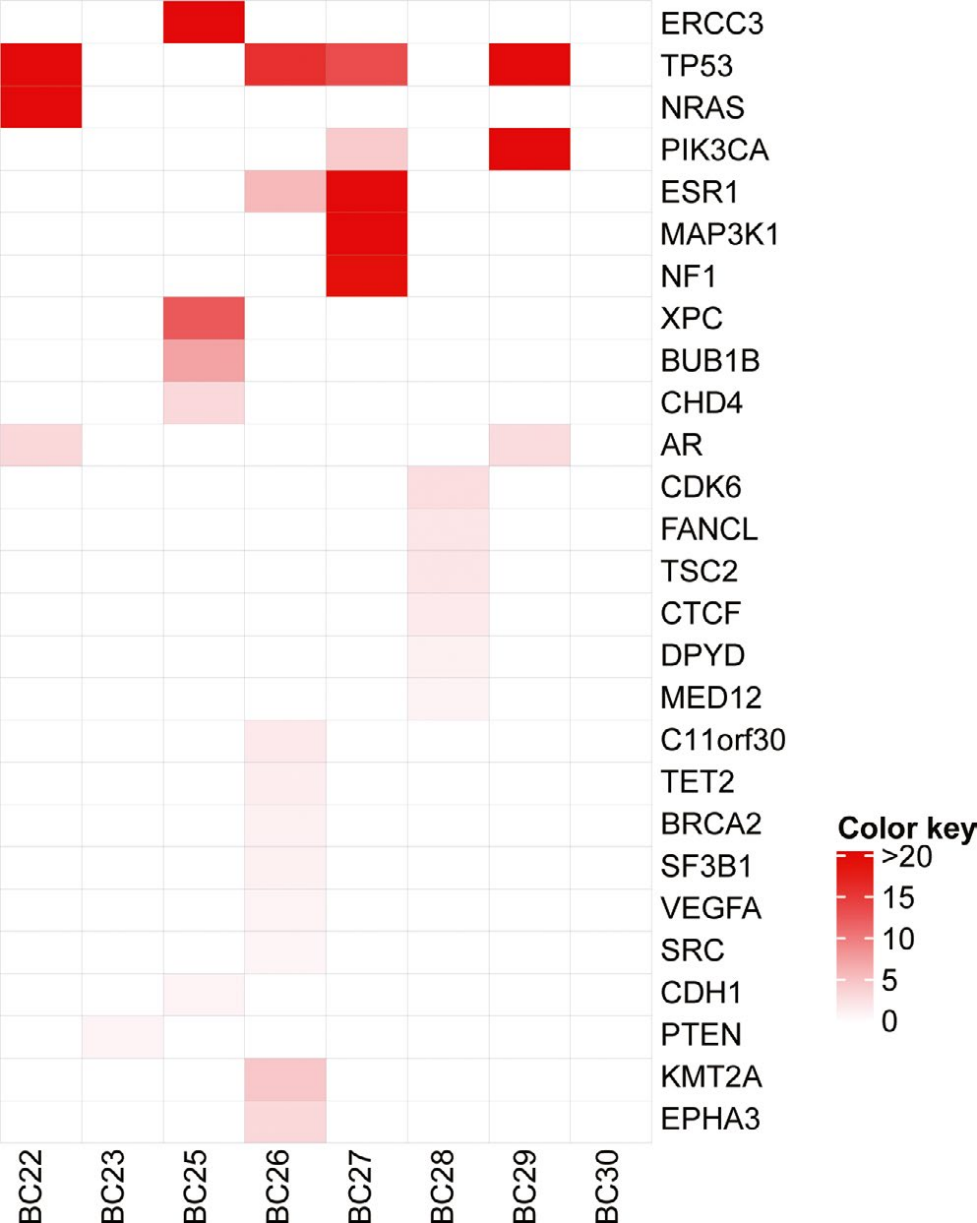
Chemotherapy resistance‐related somatic mutations observed in ctDNA profiling in HER2‐ BC patients

For the HER2‐ BC patients who received only chemotherapies and developed PD, we explored the mechanism underlying chemotherapy resistance. Since our cohort of HER2‐ BC patients was relatively small, individual patient had different somatic mutation profiling in ctDNA samples (Figure 5). Most frequent mutated genes were those well‐defined ones such as tumor suppressors *TP53, NF1,* and *MAP3K1*, oncogenes *PIK3CA* and *NRAS* (OMIM 164790)*,* and DNA damage repair genes *XPC* (OMIM 613208) and *BUB1B* (OMIM 602860). Two patients displayed *ESR1* missense mutations in their ctDNA samples: *ESR1* p.(Tyr537Ser) and p.(Asp538Gly) hotspot mutations. These two mutations have been reported as activating mutations that confer endocrine resistance (Fanning et al., 2016; Toy et al., 2017). Follow‐up study with one patient who had the *ESR1* hotspot mutation did show that this patient progressed to PD after fulvestrant endocrine therapy. Therefore, our data imply that ctDNA might be used to detect chemotherapy‐resistant mutations and predict the drug response in HER2‐ BC patients, encouraging future studies of examining the drug‐resistant mechanism in bigger patient cohorts.

## 4 DISCUSSION

In the current study, we identified multiple ctDNA mutations in HER2+ BC patients with IR and AR to trastuzumab and in HER2‐ BC patients with resistance to chemotherapy. We found that HER2+ BC patients who benefited from trastuzumab had lower levels of *ERBB2* somatic copy number when examined in ctDNA samples, compared to HER2+ BC patients who had PD. This has potential clinical relevance because doctors can monitor patients' response to targeted drugs via a noninvasive real‐time liquid biopsy approach. Moreover, somatic mutation profiling from serial plasma sampling can indicate potential deleterious mutations which can predict patients' prognosis and their development of drug resistance. Given that these results were obtained with a small number of patients, they need to be further validated in larger patient cohorts.

The introduction of trastuzumab therapy markedly improved the prognosis associated with HER2+ BC. Despite this, the presence of IR and AR to trastuzumab treatment remains a significant challenge. It is proposed that several mechanisms may contribute to trastuzumab resistance: (a) obstacles preventing trastuzumab binding to HER2, such as p95 truncating mutation (Scaltriti et al., 2007); (b) upregulation of HER2 downstream signaling pathways, such as activating mutation of oncogene PI3K or loss of functions of negative regulator PTEN (Berns et al., 2007; Nagata et al., 2004); (c) signaling through alternate pathways, such as insulin‐like growth factor I receptor pathway or other EGFRs (Huang et al., 2010; Nahta, Yuan, Zhang, Kobayashi, & Esteva, 2005); and (d) failure to trigger an immune‐mediated mechanism to destroy tumor cells (Musolino et al., 2008; Pohlmann, Mayer, & Mernaugh, 2009). In our study, using NGS to identify resistance‐related genes in the ctDNA from liquid biopsies, we found that activating mutations in *PIK3CA* p.(Cys420Arg; patient BC2), mutations in *TP53* (all three AR patients), and missense mutation in *ERBB2* p.(Leu869Arg; patient BC1) could contribute to trastuzumab resistance and the mutation profile for patients with IR and AR to trastuzumab could be different, although the results need to be further investigated and validated.

Although ctDNA profiling potentially provides valuable information to dissect the candidate genomic alterations associated with trastuzumab or chemotherapy resistance, this technique does have several limitations before full implementation into clinical care (Heitzer, Haque, Roberts, & Speicher, 2018). A better sequencing coverage depth and more comprehensive computational tools will improve our understanding of ctDNA and its application in the clinics. Together, our retrospective study reported a great potential of using ctDNA mutation profiling to identify trastuzumab and chemotherapy resistance‐related genes. Further studies in larger patient cohorts with matched pretreatment samples may be of interest to study the resistance mechanisms.

## CONFLICT OF INTEREST

Yang W. Shao, Xue Wu, Ruoying Yu, and Tian Sun are the shareholders or employees of Geneseeq Technology Inc. Junrong Yan is the employee of Nanjing Geneseeq Technology Inc.

## Data Availability

The data that support the findings of this study are available from the corresponding author upon reasonable request.
