# Supplementary figures and images for: Monitoring treatment efficacy and resistance in breast cancer patients via circulating tumor DNA genomic profiling
Source: Mol Genet Genomic Med. 2019 Dec 23;8(2):e1079. doi: 10.1002/mgg3.1079 (PMC7005625; doi:10.1002/mgg3.1079)

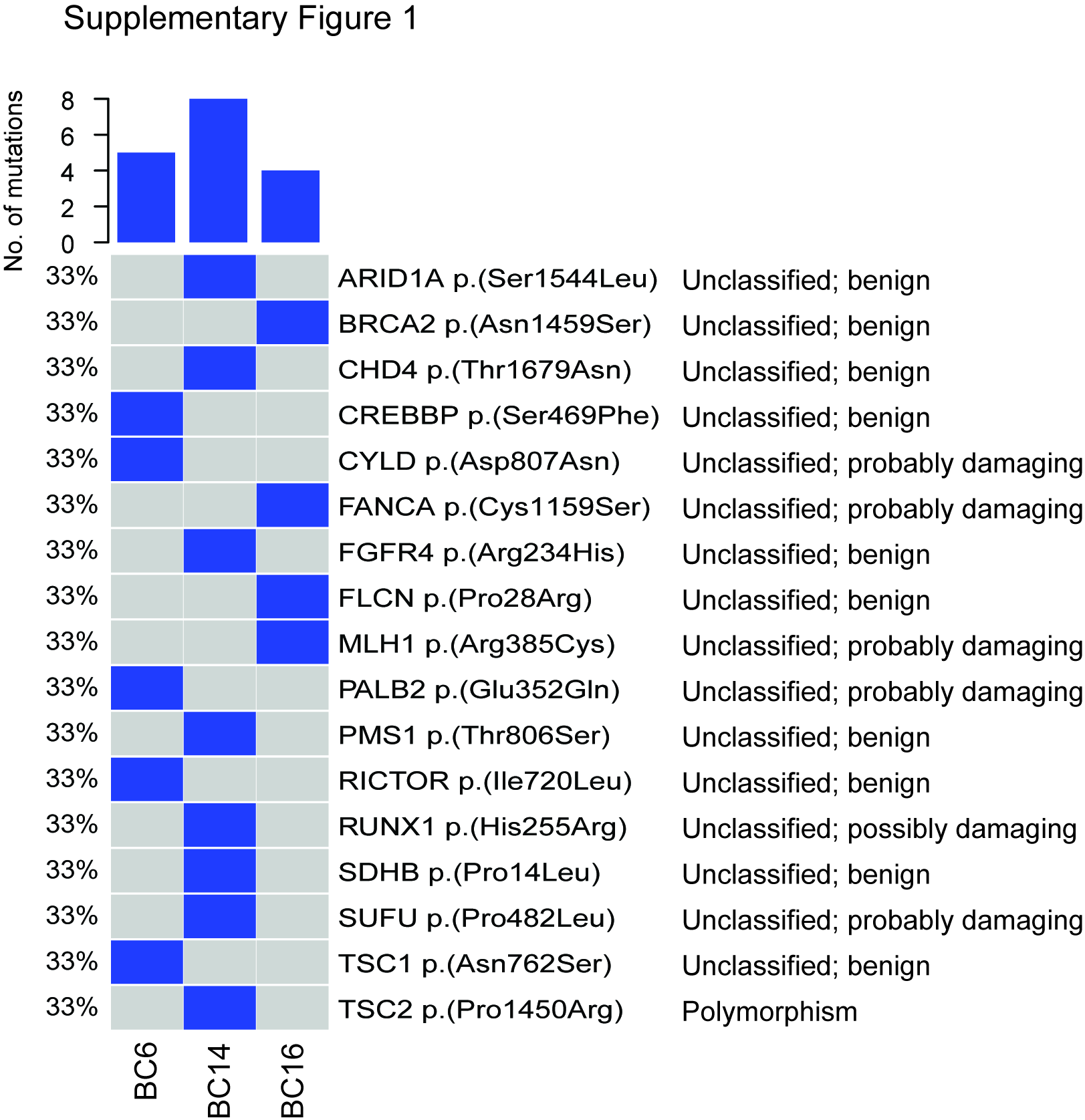

Supplement: Supplementary file 1 [file MGG3-8-e1079-s001.tif]

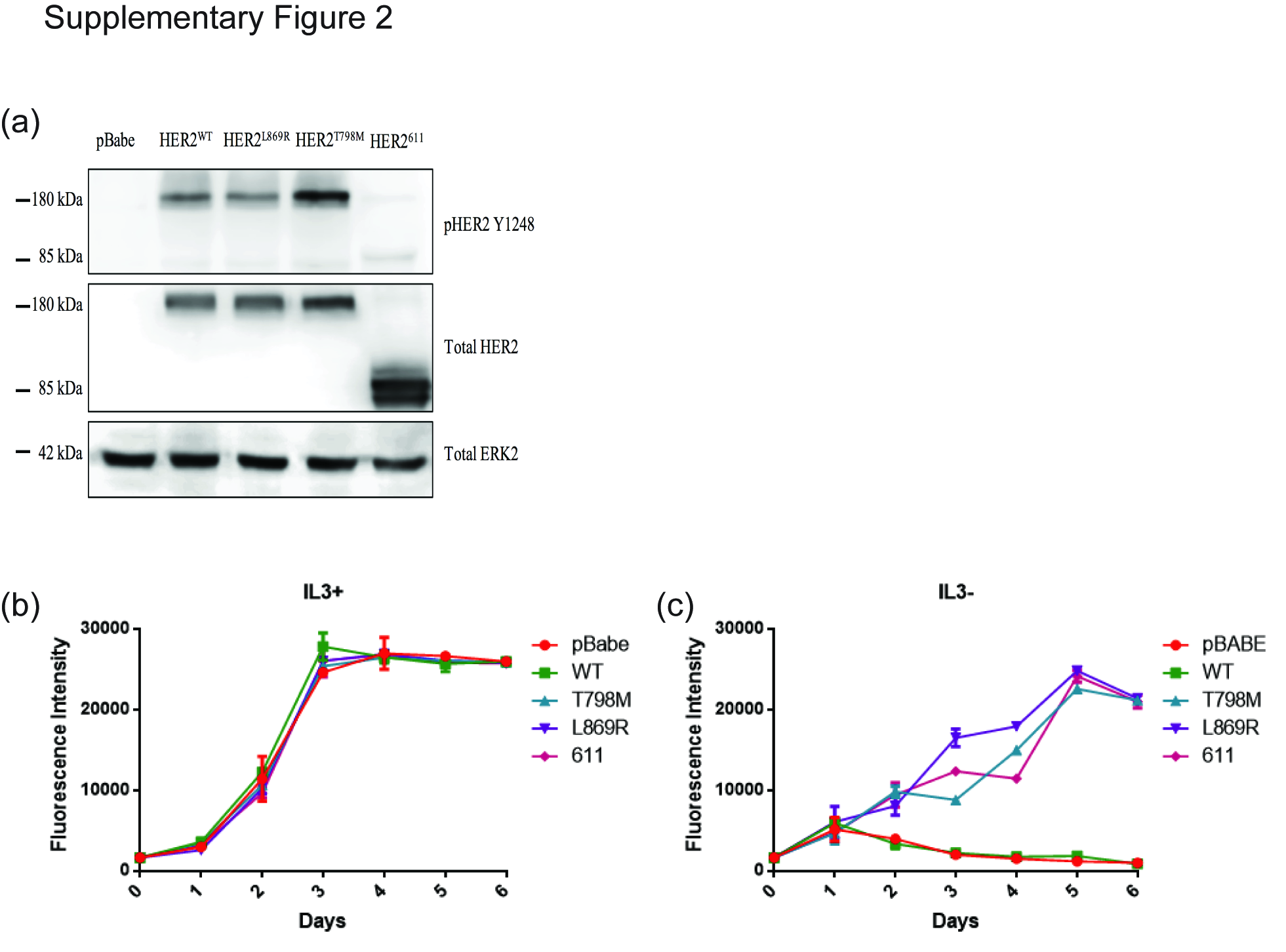

Supplement: Supplementary file 2 [file MGG3-8-e1079-s002.tif]
